# Supplementary material for: Anti-Aging Effect of Adipose-Derived Stem Cells in a Mouse Model of Skin Aging Induced by D-Galactose
Source: PLoS One. 2014 May 15;9(5):e97573. doi: 10.1371/journal.pone.0097573 (PMC4022592; doi:10.1371/journal.pone.0097573)
Supplement: File S1 — Ethics statement. (DOCX) [file pone.0097573.s001.docx]

**ETHICS STATEMENT**

The animal experimental protocols were approved by the Southern Medical University Laboratory Animal Administration Committee, and performed according to the Southern Medical University Guidelines for Animal Experimentation. All efforts were made to minimize suffering.
