# Supplementary material for: Anti-Aging Effect of Adipose-Derived Stem Cells in a Mouse Model of Skin Aging Induced by D-Galactose
Source: PLoS One. 2014 May 15;9(5):e97573. doi: 10.1371/journal.pone.0097573 (PMC4022592; doi:10.1371/journal.pone.0097573)
Supplement: File S2 — Certificate for proofreading by Bioedit. (DOCX) [file pone.0097573.s002.docx]

**Title:** Anti-aging effect of adipose-derived stem cells in a mouse model of skin aging induced by D-galactose

**Authors:** Shengchang Zhang, MD, Ziqing Dong, MD, Zhangsong Peng, MD, Feng Lu, MD, PhD

Shengchang Zhang and Ziqing Dong contributed equally and are co-first authors

**Institution:** Department of Plastic and Cosmetic Surgery, Nanfang Hospital, Southern Medical University, Guang Zhou, Guang Dong, P.R, China

**Corresponding author:** Feng Lu, MD, PhD

Tel/Fax: +86-020-61641869

E-mail address: doctorlufeng@hotmail.com

**Key words:** adipose stem cells, anti-aging, advanced glycation end products

**ABSTRACT**

**Introduction:** Glycation products accumulate during aging of slowly renewing tissue, including skin, and are suggested as an important mechanism underlying the skin aging process. Adipose-derived cells are widely used in the clinic to treat ischemic diseases and enhance wound healing. Interestingly, adipose-derived stem cells (ASCs) are also effective in anti-aging therapy, although the mechanism underlying their effects remains unknown. The purpose of the present study was to examine the anti-aging effect of ASCs in a D-galactose-induced aging animal model and to clarify the underlying mechanism.

**Materials & methods:** Six-week-old nude mice were subcutaneously injected with D-gal daily for 8 weeks. Two weeks after completion of treatment, mice were randomized to receive subcutaneous injections of 10^6^ green fluorescent protein-expressing ASCs aminoguanidine (AG) or phosphate-buffered saline (PBS). Control mice received no treatment. We examined tissue histology and determined the activity of senescence-associated molecular markers such as superoxide dismutase (SOD) and malondialdehyde (MDA).

**Results:** Transplanted ASCs were detectable for 28 days after injection. ASCs inhibited advanced glycation end product (AGE) levels in our animal model as well as increased the SOD level and decreased the MDA level, all of which act to reverse the aging phenotype in a similar way to AG, an inhibitor of AGE formation. Furthermore, ASCs released angiogenic factors *in vivo* such as vascular endothelial growth factor, suggesting a skin trophic effect.

**Conclusions:** These results demonstrate that ASCs may contribute to the regeneration of skin during aging. In addition, the data shows that ASCs provide a functional benefit by glycation suppression, antioxidation, and trophic effects in a mouse model of aging.

**Introduction**

Aging is a biological process that induces changes to the structural integrity and physiological function of skin [1], such as the development of dyschromia, roughness, and fine rhytids followed by persistent deeper folds. Structural changes are a result of dermal atrophy, decreased collagen, the loss of subcutaneous fat, the loss of inherent elasticity, and increased melanogen [2]. Several theories have been proposed to explain this process, including the accumulation of genomic mutations, the accumulation of toxic metabolites, hormonal deprivation, the increased formation of free radicals (oxidative damage), and the cross-linking of macromolecules under glycation [3].

Glycation is a nonenzymatically driven reaction between free amine groups, such as amino acids in proteins, and reducing sugars like glucose. This reaction, also called the Maillard reaction, eventually leads to the formation of advanced glycation end products (AGEs) such as carboxymethy l-L-Lysine and pentosidine, which may be responsible for cross-linking between macromolecules through covalent bonding. Glycation most commonly occurs in tissues in which macromolecular structures have a slow turnover rate and is therefore thought to play an important role in aging [4]. Accumulating evidence indicates that AGEs exacerbate and accelerate the aging process and contribute to the early phases of age-related diseases, including neurodegenerative disease, cataracts, renal failure, arthritis, and age-related macular degeneration [5, 6]. Moreover, AGEs and their precursors usually contain reactive carbonyl groups generated by reactive oxygen species (ROS) [7, 8]. ROS bind to polyunsaturated lipids, forming malondialdehyde (MDA), which is a reactive aldehyde and one of many reactive electrophile species that causes toxic stress in cells similarly to AGEs. Therefore, the level of MDA could be used as a marker of the aging process [9]. Superoxide dismutases (SOD) are enzymes that catalyze the dismutation of superoxide into oxygen and hydrogen peroxide and play an important role in antioxidant defense in nearly all cells exposed to oxygen. For these reasons, the expression of SOD may be another marker related to the aging process.

Other than glycation, alterations in skin collagen content and dermal vascularization also play key roles in aging. As the process of aging advances, collagen fibers become thinner, thereby changing the collagen proportion in tissues. In fact, with advanced age, collagen fibers in the deep dermis undergo lysis and become thinner. Moreover, a progressive reduction in dermis vasculature is also seen, resulting from a reduction in the number and size of vascular vessels, which is associated with alterations in vascular wall components and other changes that progress until the vessels are no longer functional [10, 11].

Previous studies indicated that adipose tissue transplantation could improve skin quality at the recipient site in addition to increase skin volume [12, 13]. This unexpected consequence of adipose tissue transplantation may be due to the effect of mesenchymal stem cells (MSCs) in the stromal-vascular fraction of subcutaneous adipose tissue, or adipose-derived stem cells (ASCs). ASCs exhibit multi-lineage developmental plasticity and are similar to bone-marrow-derived MSCs in terms of surface markers and gene profiling [14, 15]. In addition, many clinical studies and animal experiments have conﬁrmed that the injection of these cells has favorable effects on wound repairing, immunomodulation, and anti-apoptosis via a paracrine effect or differentiation [16, 17]. Moreover, recent studies also revealed that ASCs improve wrinkles resulting from photo-aging and promote collagen synthesis and epidermal thickening of photo-aged ﬁbroblasts *in vitro* [18].

However, the underlying mechanisms of the anti-aging effects of ASCs have not been extensively studied. Therefore, in an attempt to further understand these mechanisms, we designed an experimental animal study of skin aging induced by D-galactose (D-gal). The goal of this study was to use histologic and immunohistologic analyses to assess the anti-aging effects of ASCs, especially in the suppression of glycation and restoration of functional capacity.

**Materials and methods**

**Ethics statement**

Animal experimental protocols were approved by the Southern Medical University Laboratory Animal Administration Committee, and experiments were performed according to the Southern Medical University Guidelines for Animal Experimentation. All efforts were made to minimize animal suffering.

**Isolation and culture of ASCs**

Mouse inguinal fat pad adipose tissue samples were acquired from 6-week-old green fluorescent protein (GFP)-expressing mice, which were provided by the Model Animal Research Center of Nanjing University (Nanjing, China). The obtained samples were cut into pieces and digested with 0.075% type I collagenase (Sigma–Aldrich, St. Louis, MO) under gentle agitation for 45 min at 37°C. Mature adipocytes and connective tissue cells were separated from pellets by centrifugation (800 *g* for 10 min) and then discarded. The pellets were resuspended in phosphate-buffered saline (PBS) and filtered through a 200 μm mesh followed by centrifugation (800 *g* for 10 min) to spin down stromal-vascular fraction cell pellets. The retrieved cell fraction was cultured overnight at 37°C with 5% CO_2_ in a control medium (Dulbecco’s modified Eagle media, 10% fetal bovine serum, 100 units/mL penicillin, 100 mg/mL streptomycin). The resulting cell population was cultured for 3 to 5 days until confluent. ASCs were cultured and expanded in the control medium. Cells from P3 to P5 were used in the following experiments.

**Differentiation of mouse ASCs**

*In vitro* multi-lineage differentiation of ASCs was induced in the control medium supplemented with one of the three formulas described below, as previously described [19]. *In vitro*-cultured ASCs were detected using Oil-red O, Alizarin red, and Alcian blue staining, which identified fat, bone, and cartilage cells, respectively, differentiated from ASCs.

**D-galactose (D-gal)-induced aging model and animal experiments**

Chronic administration of a low dose of D-gal has been widely used as an animal model for aging in studies of skin aging or anti-aging pharmacology [20]. In this model, the AGE inhibitor aminoguanidine (AG) prevents aging phenotypes, suggesting AGEs as a pivotal player in the underlying mechanism of aging [21].

A total of 80 6-week-old nude mice (gender not considered) were provided by the Southern Medical University Experimental Animal Center (Guangzhou, China). Mice were randomly divided into four groups (n=20 each). Three groups of animals received daily subcutaneous injections of D-gal (1,000 mg/kg, subcutaneously) for 8 weeks. Two weeks later, animals of these three groups received a subcutaneous injection of 10^6^ GFP-expressing ASCs, AG (100 mg/kg, intragastrically), or PBS at the midline of the dorsum. After the injection, all four groups of mice were housed for another four weeks. All animals were allowed free access to water and a chow diet and were observed daily. Mice were sacrificed at the end of treatment, and skin tissue was immediately collected or stored at -70°C until further use.

**Survival of ASCs**

After injection of GFP-expressing ASCs, mice were anesthetized with isoflurane and underwent fluorescence live imaging using the Kodak *In-Vivo* Imaging System F (Carestream Health, Inc. Rochester, NY, US) at days 1, 3, 7, 14, and 28 after injection.

**Histological examination**

Skin tissue from animals of all four groups were fixed in 4% paraformaldehyde, dehydrated, and paraffin-embedded for haematoxylin and eosin (H&E) staining. Tissue blocks were serially sectioned (6 µm sections), mounted onto an APES-treated glass slide, assessed under an Olympus BX51 microscope, and photographed using an Olympus DP71 digital camera. The dermal thickness of the skin samples was measured.

**Collagen quantification**

To determine the amount of total collagen, samples obtained from all four groups were stained with Masson’s trichrome. Sections were deparaffinized in xylene, rehydrated in graded ethanol, and post-fixed in Bouin's fixative for 1 h at 55°C. The nuclei were stained with an equal volume of ferric chloride solution, and then collagen was stained with an alcoholic hematoxylin and trichrome solution. Total collagen content was reported as a percentage of the aniline blue staining divided by the total tissue area of the section using the Image J software.

**Immunohistochemistry of CD31 and vascular endothelial growth factor (VEGF)**

Immunohistochemistry was used to detect angiogenesis in the samples. Sections obtained from each group were stained with an anti-CD34 antibody (Abcam, Cambridge, UK) and an anti-VEGF antibody (Abcam, Cambridge, UK). Paraffin sections were dewaxed and hydrated before immunohistochemical staining. Slides were washed with PBS, incubated in 3% H_2_O_2_ for 10 min, washed once more in PBS, and then incubated in a protein block solution for 30 min. Then, sections were incubated with the primary antibody at 4°C overnight. The next day, the sections were washed three times and then incubated with a biotinylated secondary antibody. After a 30-min incubation with a complex of avidin and biotinylated horseradish peroxidase, the enzyme activity was visualized using 3,3' -diaminobenzidine. Slides were scored by two independent observers using an Olympus BX51 microscope and photographed with the use of an Olympus DP71 digital camera. The number of CD31-positive vessels was counted and the VEGF-positive area was quantified using the Sigma Scan software on five nonconsecutive tissue sections for each image.

**Measurement of superoxide dismutase activity and lipid peroxidation**

**Tissue homogenization**

Skin tissue samples were weighed and homogenized in normal saline to generate 5% homogenates. Homogenates were sonicated twice at 30 s intervals. Homogenization and sonication were performed at 4°C. After sonication, homogenates were sequentially centrifuged at 3,000 rpm for 10 min and 12,000 rpm for 15 min. Aliquots of supernatants were used for further experiments. The protein content of the aliquots was determined using a BCA protein assay kit (Pierce Chemical Co.).

SOD activity of the skin was examined using the xanthine oxidase method with a commercial kit (Nanjing Jiancheng Bioengineering Institute, China), as previously described [21]. This assay involves a xanthine-xanthine oxidase system that reacts with 2-(4-iodophenyl)-3-(4-nitrophenol-5-phenlyltet-razolium chloride) to form a red formazan dye at an absorbance at 550 nm and produces superoxide ions. The protein concentration was determined using a BCA protein assay kit (Pierce Chemical Co.), with one unit of SOD defined as the amount of SOD inhibiting the rate of reaction by 50% at 25°C.

Lipid peroxidation was evaluated by assessing the MDA content using a thiobarbituric acid (TBA) method as recommended (Nanjing Jiancheng Bioengineering Institute, China). This method is based on the spectrophotometric measurement of color produced during the MDA reaction with TBA. MDA concentrations were calculated through the absorbance of TBA reactive substances (TBARS) at 532 nm.

**Inhibition of x AGEs** **formation *in vitro***

AGE-modified bovine serum albumin (BSA) was prepared, as previously described [22]. Briefly, BSA (100 mg/mL) was incubated under sterile conditions with 0.5 M D-gal in 0.2 M PBS (pH 7.4) at 37°C for 8 weeks. For ASC treatment or AG inhibition, AGE-modified BSA samples were incubated with ASCs (1 × 10^6^) or AG (100 mm), respectively, under identical conditions. A control BSA sample was incubated under identical conditions but without D-gal. Samples were dialyzed (10 kDa cut-off) against PBS, and the BSA-AGEs content was determined using a commercial ELISA kit, as previously described [23].

**Statistical analysis**

Statistical analyses were performed using SPSS 13.0 (SPSS Inc., Chicago, IL). Results were compared using ANOVA, with post-hoc LSD test as appropriate. A P-value of < 0.05 was considered statistically significant. Data are shown as mean ± SD.

**Results**

**Characterization of ASCs**

ASCs expanded easily *in vitro* and showed fibroblast-like morphologic features (Fig. 1A). To verify their multipotent differentiation, ASCs were incubated in media known to induce an adipogenic, osteogenic, or chondrogenic lineage. Adipogenic differentiation was determined by Oil Red O staining of intracellular lipid droplets (Fig. 1B), osteogenic differentiation through Alizarin red S staining of matrix mineralization (Fig. 1C), and chondrogenic differentiation through Alcian blue staining of cartilage-specific proteoglycans (Fig. 1D).

**Retention rate of transplanted ASCs in a mouse aging model**

GFP signals were detected in fluorescence live imaging of mice throughout the experiment (Fig. 2A). Signals were limited to the dorsum at day 1, with the injection site showing the strongest signal. At day 3, the intensity of signals at the injection area decreased but remained strong. From days 7 to 14, the intensity of signals in the dorsum area decreased and was weak by day 28 (Fig. 2B).

**Effect of ASCs on the formation of AGEs in mice**

After ASC treatment, visual inspection revealed no major abnormalities in mice. Mice of all groups gained weight normally throughout the study (Fig. 3A). As expected, mice treated with D-gal showed a remarkably increased level of skin AGEs compared to the control group (P < 0.05) (Fig. 3B), and AG treatment significantly reversed the increased level of AGEs in D-gal-treated mice (P < 0.05) (Fig. 3B). Similar to the effect of AG, ASC treatment was effective in significantly blocking the increase in the AGEs level (P < 0.05) (Fig. 3B), suggesting that ASCs have an inhibitory effect on BSA-AGE formation.

**Effect of ASCs on antioxidant enzyme activity and lipid peroxidation in mice**

To further conﬁrm that ASCs have a protective effect on the skin by antioxidant action, we measured the levels of SOD and MDA, an indicator of lipid peroxidation, in mouse skin tissue. As expected, SOD levels decreased, while MDA levels significantly increased; however, treatment with ASCs increased the SOD and decreased the MDA expression levels in D-gal-treated mouse skin (Figs. 3C and D).

**Histological observation**

H&E staining showed significant changes in skin appendages in samples from D-gal-treated mice compared to control mice (Fig. 4A). Moreover, dermal thickness was signiﬁcantly lower in D-gal-treated mice and significantly higher in the ASC-treated group compared to that of the control group (Fig. 4B). Quantification of collagen content showed that the ASC-treated group also had a higher collagen content than the D-gal-treated group (Fig. 4C).

**ASCs increase VEGF levels and enhance skin** **tissue angiogenesis**

To further confirm that ASCs induce skin angiogenesis, we measured CD31-positive microvessels and VEGF expression in skin tissue (Fig. 5A). As expected, the ASC-treated group had higher microvessel density and VEGF expression levels than the D-gal-treated group (Figs. 5B and C).

**Discussion**

Stem cells have various potential uses in most medical areas due to their differentiation and paracrine effects. In particular, ASCs have several advantages in clinical applications because they are easy to harvest and abundant in the human body, meaning that there are no ethical problems in harvesting these cells. In this study, we examined the anti-aging effects of ASCs, particularly focusing on the suppression of the glycation reaction and restoration of the functional capacity of skin in a mouse model of accelerated aging induced by D-gal. Our findings can be summarized as follows: (1) ASCs can survive up to 28 days after being injected into dermal tissue; (2) ASCs can decrease the AGE level, therefore reversing the aging phenotype, which is a similar effect to that of AG, and inhibitors of AGEs and ASCs can decrease the expression of senescence-associated markers such as SOD and MDA; (3) ASCs can significantly increase dermal thickness and collagen content of the skin; and (4) ASCs can increase the expression level of VEGF and increase the vessel density of the skin, indicating a possible skin trophic effect of ASCs.

Skin aging occurs through intrinsic and extrinsic pathways. Intrinsic aging, so-called normal aging, is conﬁrmed by changes in the levels of senescence-associated molecular markers. A previous study demonstrated that D-gal injection leads to an accelerated aging phenotype, as well as changes in AGE level and in the expression levels of senescence markers such as SOD and MDA [24, 25]. In our study, nude mice treated with D-gal showed signiﬁcant changes resembling normal aging. In addition, the AGE inhibitor, AG, prevented the accelerated aging process. These results strongly suggest that AGEs are a crucial mediator in our D-gal-induced aging model.

An important barrier in cell therapy remains the low engraftment rate of transplanted cells, which diminishes the efficiency of cell therapy [26]. Previous studies suggested that transplanted ASCs have very low retention in the later stages of the transplant [27, 28, 29]. Remarkably, in our study, the GFP signal of injected ASCs was detectable until day 28. The low survival rate of transplanted ASCs may be a result of phagocytosis by local immune cells. However, transplanted cells cannot be entirely responsible for the beneficial effect on aging; the paracrine effect is more likely to be the mechanism explaining the functional results [30].

Previous studies have suggested that excess AGE intake and chronic accumulation of AGE-related glycated proteins in tissue may further potentiate the aging process, resulting in impaired mitochondrial function and decreased life span in *Caenorhabditis elegans* and mice [31, 32, 33]. Our results showed that ASC treatment inhibits AGE formation and that ASCs had less of an inhibitory effect on the formation of AGEs than AG, suggesting that ASC treatment inhibits BSA-AGE formation. Cells in the body possess a wide range of inter-linked antioxidant defense mechanisms to protect themselves against damage caused by ROS. Among these mechanisms, antioxidant enzymes, including SODs, are important in scavenging remaining ROS in cells. SODs are metalloenzymes that catalyze the dismutation of superoxide anion to molecular O_2_ and H_2_O_2_ and are, therefore, a crucial part of the cellular antioxidant defense mechanism. SODs exist in cells and tissues in three forms, namely, cytosolic Cu/Zn-SOD (SOD1), mitochondrial Mn-SOD (SOD2), and extracellular SOD (SOD3). Cu/Zn-SOD and Mn-SOD are thought to be important in defense against oxygen toxicity [34]. Proteomic analysis revealed 112 proteins, including SODs, which are upregulated by ASC treatment. Representative proteins showed antioxidant effects on epithelial cells in previous studies [35, 36, 37]. An important finding of the present study is that ASCs reversed the effects of D-gal-induced oxidative stress in mouse skin, as shown by the expression levels of senescence-associated molecular markers such as SOD and MDA. Secretary proteins from ASCs such as SOD and several cytokines may mediate the protective effects and play key roles *in vivo*. Stem cells may possess potent antioxidant effects as suggested by the decrease in expression level of senescence-associated molecular markers following ASC treatment.

Kim et al. suggested that ASC-conditioned medium enhanced type 1 collagen secretion and fibroblast migration of human dermal fibroblasts in an *in vitro* wound-healing model [38]. In another study, wrinkles induced by UVB irradiation were significantly improved by subcutaneous injection of adipose-derived stem cells (ADSCs) in hairless mice. In addition, dermal thickness and collagen content were higher in animals of ASC-injection groups than of control groups [18]. Our histological observations showed lower expression levels of collagen and dermal thickness in D-gal-induced mice, which was increased by ASC injection. While some studies suggested that ASCs secret collagen [39, 40], the low retention rate of ASCs indicate that the increasing collagen expression level was more likely due to the upregulation of collagen expression in local fibroblasts caused by paracrine ASCs.

Another potential role of ASC treatment in skin anti-aging is angiogenesis. Substantial evidence indicates that ASCs may increase angiogenesis through secretion of angiogenic factors such as VEGF and hepatocyte growth factor [41, 42, 43]. In this study, our results support the notion that ASC transplantation strongly induces the revascularization of skin tissue along with the secretion of VEGF, which was clearly detected in transplanted cells in our model, thereby lending support to the trophic hypothesis. CD31 staining studies further confirmed this hypothesis. Although previous studies indicated that ASCs can differentiate into vascular endothelial cells [44, 45], we found that GFP-positive ASCs were undetectable after 28 days, indicating that angiogenesis was mainly a result of a paracrine effect of ASCs.

**CONCLUSIONS**

In summary, we examined the glycation suppression of ASCs in a mouse aging skin model induced by D-gal. ASCs may have the potential to contribute to the regeneration of skin and provide a functional benefit. ASC injection prevented the expression of senescence-associated molecular markers. Similarly to AG, the inhibitor of AGE formation, ASCs inhibited D-gal-increased AGE levels, therefore reversing the aging phenotype in our mouse model. The expression levels of SOD and MDA in skin were increased with ASC injection, suggesting that ASCs may suppress glycation in skin. ASCs may be a good candidate for the control and prevention of skin damage caused by glycation in various skin conditions, including wounding and aging.

**Acknowledgments**

This work was supported financially by the National Nature Science Foundation of China (81171834, 81071589), and the Medical Scientific Research Foundation of Guangdong Province (A2013449, A2011453).

**References**

1. Farage MA, Miller KW, Elsner P, Maibach HI (2008) Functional and physiological characteristics of the aging skin. Aging Clin Exp Res 20: 195-200.

2. Glogau RG (1997) Physiologic and structural changes associated with aging skin. Dermatol Clin 15: 555-559.

3. Johnson FB, Sinclair DA, Guarente L (1999) Molecular biology of aging. Cell 96: 291-302.

4. Frye EB, Degenhardt TP, Thorpe SR, Baynes JW (1998) Role of the Maillard reaction in aging of tissue proteins. Advanced glycation end product-dependent increase in imidazolium cross-links in human lens proteins. J Biol Chem 273: 18714-18719.

5. Tian J, Ishibashi K, Ishibashi K, Reiser K, Grebe R, et al. (2005) Advanced glycation endproduct-induced aging of the retinal pigment epithelium and choroid: a comprehensive transcriptional response. Proc Natl Acad Sci U S A 102: 11846-11851.

6. Semba RD, Nicklett EJ, Ferrucci L (2010) Does accumulation of advanced glycation end products contribute to the aging phenotype? J Gerontol A Biol Sci Med Sci 65: 963-975.

7. Chen HY, Jang S, Jinn TR, Chang JY, Lu HF, et al. (2012) Oxygen radical-mediated oxidation reactions of an alanine peptide motif - density functional theory and transition state theory study. Chem Cent J 6: 33.

8. Stadtman ER (1992) Protein oxidation and aging. Science 257: 1220-1224.

9. Gil P, Farinas F, Casado A, Lopez-Fernandez E (2002) Malondialdehyde: a possible marker of ageing. Gerontology 48: 209-214.

10. Fisher GJ, Quan T, Purohit T, Shao Y, Cho MK, et al. (2009) Collagen fragmentation promotes oxidative stress and elevates matrix metalloproteinase-1 in fibroblasts in aged human skin. Am J Pathol 174: 101-114.

11. Bonta M, Daina L, Mutiu G (2013) The process of ageing reflected by histological changes in the skin. Rom J Morphol Embryol 54: 797-804.

12. Mojallal A, Lequeux C, Shipkov C, Breton P, Foyatier JL, et al. (2009) Improvement of skin quality after fat grafting: clinical observation and an animal study. Plast Reconstr Surg 124: 765-774.

13. Coleman SR (2006) Structural fat grafting: more than a permanent filler. Plast Reconstr Surg 118: 108S-120S.

14. Zuk PA, Zhu M, Ashjian P, De Ugarte DA, Huang JI, et al. (2002) Human adipose tissue is a source of multipotent stem cells. Mol Biol Cell 13: 4279-4295.

15. Vieira NM, Brandalise V, Zucconi E, Secco M, Strauss BE, et al. (2010) Isolation, characterization, and differentiation potential of canine adipose-derived stem cells. Cell Transplant 19: 279-289.

16. Kilroy GE, Foster SJ, Wu X, Ruiz J, Sherwood S, et al. (2007) Cytokine profile of human adipose-derived stem cells: expression of angiogenic, hematopoietic, and pro-inflammatory factors. J Cell Physiol 212: 702-709.

17. Meliga E, Strem BM, Duckers HJ, Serruys PW (2007) Adipose-derived cells. Cell Transplant 16: 963-970.

18. Kim WS, Park BS, Park SH, Kim HK, Sung JH (2009) Antiwrinkle effect of adipose-derived stem cell: activation of dermal fibroblast by secretory factors. J Dermatol Sci 53: 96-102.

19. Sen A, Lea-Currie YR, Sujkowska D, Franklin DM, Wilkison WO, et al. (2001) Adipogenic potential of human adipose derived stromal cells from multiple donors is heterogeneous. J Cell Biochem 81: 312-319.

20. Wei H, Li L, Song Q, Ai H, Chu J, et al. (2005) Behavioural study of the D-galactose induced aging model in C57BL/6J mice. Behav Brain Res 157: 245-251.

21. Huebschmann AG, Regensteiner JG, Vlassara H, Reusch JE (2006) Diabetes and advanced glycoxidation end products. Diabetes Care 29: 1420-1432.

22. Makita Z, Vlassara H, Cerami A, Bucala R (1992) Immunochemical detection of advanced glycosylation end products in vivo. J Biol Chem 267: 5133-5138.

23. Kaida Y, Fukami K, Matsui T, Higashimoto Y, Nishino Y, et al. (2013) DNA aptamer raised against AGEs blocks the progression of experimental diabetic nephropathy. Diabetes 62: 3241-3250.

24. Mao GX, Deng HB, Yuan LG, Li DD, Li YY, et al. (2010) Protective role of salidroside against aging in a mouse model induced by D-galactose. Biomed Environ Sci 23: 161-166.

25. Song X, Bao M, Li D, Li YM (1999) Advanced glycation in D-galactose induced mouse aging model. Mech Ageing Dev 108: 239-251.

26. Cassino TR, Drowley L, Okada M, Beckman SA, Keller B, et al. (2012) Mechanical loading of stem cells for improvement of transplantation outcome in a model of acute myocardial infarction: the role of loading history. Tissue Eng Part A 18: 1101-1108.

27. Mazo M, Cemborain A, Gavira JJ, Abizanda G, Arana M, et al. (2012) Adipose stromal vascular fraction improves cardiac function in chronic myocardial infarction through differentiation and paracrine activity. Cell Transplant 21: 1023-1037.

28. Lee TJ, Bhang SH, Yang HS, La WG, Yoon HH, et al. (2012) Enhancement of long-term angiogenic efficacy of adipose stem cells by delivery of FGF2. Microvasc Res 84: 1-8.

29. Bhang SH, Cho SW, Lim JM, Kang JM, Lee TJ, et al. (2009) Locally delivered growth factor enhances the angiogenic efficacy of adipose-derived stromal cells transplanted to ischemic limbs. Stem Cells 27: 1976-1986.

30. Ramos GA, Hare JM (2007) Cardiac cell-based therapy: cell types and mechanisms of actions. Cell Transplant 16: 951-961.

31. Morcos M, Du X, Pfisterer F, Hutter H, Sayed AA, et al. (2008) Glyoxalase-1 prevents mitochondrial protein modification and enhances lifespan in Caenorhabditis elegans. Aging Cell 7: 260-269.

32. Schlotterer A, Kukudov G, Bozorgmehr F, Hutter H, Du X, et al. (2009) C. elegans as model for the study of high glucose- mediated life span reduction. Diabetes 58: 2450-2456.

33. Sell DR, Kleinman NR, Monnier VM (2000) Longitudinal determination of skin collagen glycation and glycoxidation rates predicts early death in C57BL/6NNIA mice. FASEB J 14: 145-156.

34. Liochev SI, Fridovich I (1997) How does superoxide dismutase protect against tumor necrosis factor: a hypothesis informed by effect of superoxide on "free" iron. Free Radic Biol Med 23: 668-671.

35. Rehman J, Traktuev D, Li J, Merfeld-Clauss S, Temm-Grove CJ, et al. (2004) Secretion of angiogenic and antiapoptotic factors by human adipose stromal cells. Circulation 109: 1292-1298.

36. Baregamian N, Song J, Jeschke MG, Evers BM, Chung DH (2006) IGF-1 protects intestinal epithelial cells from oxidative stress-induced apoptosis. J Surg Res 136: 31-37.

37. Shibuki H, Katai N, Kuroiwa S, Kurokawa T, Arai J, et al. (2002) Expression and neuroprotective effect of hepatocyte growth factor in retinal ischemia-reperfusion injury. Invest Ophthalmol Vis Sci 43: 528-536.

38. Kim WS, Park BS, Sung JH, Yang JM, Park SB, et al. (2007) Wound healing effect of adipose-derived stem cells: a critical role of secretory factors on human dermal fibroblasts. J Dermatol Sci 48: 15-24.

39. Kraus A, Woon C, Raghavan S, Megerle K, Pham H, et al. (2013) Co-culture of human adipose-derived stem cells with tenocytes increases proliferation and induces differentiation into a tenogenic lineage. Plast Reconstr Surg 132: 754e-766e.

40. Desai VD, Hsia HC, Schwarzbauer JE (2014) Reversible modulation of myofibroblast differentiation in adipose-derived mesenchymal stem cells. PLoS One 9: e86865.

41. Suga H, Glotzbach JP, Sorkin M, Longaker MT, Gurtner GC (2014) Paracrine mechanism of angiogenesis in adipose-derived stem cell transplantation. Ann Plast Surg 72: 234-241.

42. Kapur SK, Katz AJ (2013) Review of the adipose derived stem cell secretome. Biochimie 95: 2222-2228.

43. Nauta A, Seidel C, Deveza L, Montoro D, Grova M, et al. (2013) Adipose-derived stromal cells overexpressing vascular endothelial growth factor accelerate mouse excisional wound healing. Mol Ther 21: 445-455.

44. Konno M, Hamazaki TS, Fukuda S, Tokuhara M, Uchiyama H, et al. (2010) Efficiently differentiating vascular endothelial cells from adipose tissue-derived mesenchymal stem cells in serum-free culture. Biochem Biophys Res Commun 400: 461-465.

45. Sahar DE, Walker JA, Wang HT, Stephenson SM, Shah AR, et al. (2012) Effect of endothelial differentiated adipose-derived stem cells on vascularity and osteogenesis in poly(D,L-lactide) scaffolds in vivo. J Craniofac Surg 23: 913-918.

**Figure legends**

**Figure 1**

*In vitro* multi-lineage differentiation potential of adipose-derived stem cells (ASCs). **A.** Green fluorescent protein-expressing mouse ASCs. **B.** Oil Red O staining. **C.** Alizarin red staining. **D.** Alcian blue staining. Scale bars = 50 μm.

**Figure 2**

The retention rate of transplanted adipose-derived stem cells (ASCs). **A.** Fluorescence live imaging of ASCs trafficking *in vivo* in aging mice. **B.** The retention rate of ASCs at days 1, 3, 7, 14, and 28 after injection.

**Figure 3**

**A.** The changes in body weight, advanced glycation end product (AGE), and level of superoxidase dismutase (SOD) and malondialdehyde (MDA) in control, D-gal-treated, D-gal plus adipose-derived stem cells-treated, and D-gal plus aminoguanidine-treated mice. **B.** The content of bovine serum albumin-AGEs. **C and D.** SOD and MDA levels in skin. Statistically significant difference, * P < 0.05 *versus* control.

**Figure 4**

Haematoxylin and eosin staining and Masson’s trichrome staining. **A.** Adipose-derived stem cells treatment increased dermal thickness and collagen ratio of mice skin. **B.** The thickness of the dermal portion of skin. **C.** Collagen ratio (collagen ﬁbers stained blue) was measured with an image analysis program. n=4. *P < 0.05, Scale bars = 100 μm.

**Figure 5**

The changes in angiogenesis in skin tissue. **A.** Immunohistochemical detection of CD31-positive microvessels (arrows) and vascular endothelial growth factor (VEGF) expression in skin tissue in control, D-gal-treated, D-gal plus adipose-derived stem cells (ASCs)-treated, and D-gal plus aminoguanidine (AG)-treated animals. CD31: Scale bar = 200 μm, VEGF: Scale bar = 100 μm. **B.** The changes in vascular density/mm^2^ in control, D-gal-treated, D-gal plus ASCs-treated, and D-gal plus AG-treated groups determined by counting the number of CD31-positive vessels within visual fields (n=8). Means ± SEM. *P < 0.05. **C.** The D-gala plus ASCs-treated showed the highest expression of VEGF among the four groups.
